# A Pragmatic Randomized Controlled Trial Targeting Perfectionism in Young Adolescents

**DOI:** 10.1007/s12310-022-09540-3

**Published:** 2022-09-06

**Authors:** Ivana Osenk, Catherine Johnson, Tracey D. Wade

**Affiliations:** Blackbird Initiative, Flinders Institute for Mental Health and Wellbeing, Flinders University, Adelaide, South Australia Australia

**Keywords:** Perfectionism, Adolescents, Universal Intervention, Anxiety

## Abstract

Perfectionism has adverse impacts on mental health and academic outcomes. We evaluated a 5-lesson classroom intervention for young adolescents delivered by teachers for impact on perfectionism, well-being, self-compassion, academic motivation and negative affect, at post-intervention and 3-month follow-up. Classes (*N* = 636 students, *M*_age_ = 13.68, *SD* = 0.60) were randomized to intervention (*n* = 343) or classes as usual (*n* = 293). Data were analyzed using linear mixed models adjusted for baseline observation and clustering. At post-intervention no differences were found between the groups. At 3-month follow-up, anxiety showed a significant increase in the control group with no increase in the intervention group (*d* = 0.23; 95% CI: 0.05, 0.40); females in the control group had a significant decrease in well-being from post-intervention to 3 month follow-up compared to the intervention group (*d* = 0.33; 95% CI: 0.08, 0.58); students with high levels of perfectionistic concerns in the intervention had significantly lower perfectionistic strivings at 3 month follow-up than the control group ((*d* = 0.34, 95% CI: 0.19, 0.49). This intervention shows promising results when delivered by teachers. Australian New Zealand Clinical Trials Registry (ANZCTR) Trial Number: ACTRN12621000457842 (April 19, 2021).

**Supplementary Information:**

The online version contains supplementary material available at 10.1007/s12310-022-09540-3.

## Introduction

Perfectionism has been defined setting excessively high standards of performance, compulsive efforts to attain these standards, measuring self-worth entirely in terms of accomplishment, and high levels of self-criticism when standards are not attained (Shafran et al., 2002). In youth and adults, two higher-order dimensions of perfectionism have been identified through factor-analytic evidence (Frost et al., 1993; Sironic & Reeve, 2015; Stornæs et al., 2019). Perfectionistic strivings refer to the propensity to set demanding high personal standards, while perfectionistic concerns relate to critical self-appraisals following failure, concerns over making mistakes, and feelings of discrepancy between one’s expectations and perceived performance.

Substantial evidence suggests both perfectionism dimensions are considered an underlying mechanism of relevance for a broad array of psychopathologies (Limburg et al., 2017; Smith et al., 2018). In youth, those high in perfectionistic concerns and perfectionistic strivings are found to be at risk of experiencing psychological distress (Hewitt et al., 2002; Stornelli et al., 2009), eating disorder symptomatology (Vacca et al., 2020), and increased sadness (Stornelli et al., 2009). Moreover, perfectionistic strivings have been found to thwart successful therapeutic outcomes (Mitchell et al., 2013). Thus, both dimensions of perfectionism are considered maladaptive in nature and have been identified as transdiagnostic risk factors for mental health difficulties that should be targeted using intervention strategies (Egan et al., 2011).

In terms of successful learning, evidence also suggests a well-established maladaptive relationship between perfectionistic concerns and academic performance (Madigan, 2019). Perfectionistic concerns have also demonstrated adverse impacts on indicators of academic success such as increased burnout and test anxiety (Osenk et al., 2020). However, the link between successful learning and perfectionistic strivings have yielded mixed findings, with studies evidencing an association with greater academic success (Madigan, 2019), while others demonstrating those with higher levels of perfectionistic strivings are at risk of higher levels of academic stress (Flett et al., 2016), burnout (Nepon et al., 2016) and procrastination (Montgomery et al., 2017).

One argument that may explain the ‘adaptive’ findings related to perfectionistic strivings and academic outcomes despite associations with psychopathology is ongoing confusion between measuring the pursuit of perfection versus the pursuit of high standards (Gaudreau, 2019; Greenspon, 2000; Osenk et al., 2020). Indeed, evidence suggests that the High Standards subscale from the Almost Perfect Scale-Revised (APS-R; Slaney et al., 2001), which is widely used in youth to measure perfectionistic strivings, may not adequately capture the ‘all or nothing’ thinking and rigid pursuit of perfection characteristic of perfectionistic strivings, and may be more representative of a flexible setting of attainable “high standards” (Blasberg et al., 2016). Evidence for this posited differentiation has been found in meta-analytic study whereby the High Standards subscale uniquely protected youth from unhelpful academic stressors, while other perfectionistic strivings measures did not share the same relationship (Osenk et al., 2020).

The adverse effects of perfectionism on mental health and successful learning, paired with the increasing incidence in youth over the last three decades (Curran & Hill, 2019), have resulted in a call for the development of universal school-based prevention programs (Egan et al., 2011). Adolescence has been identified as a key period for the development of perfectionism (Flett et al., 2002), regardless of intellectual ability (Stricker et al., 2020). However, sex differences in the structure of perfectionism, particularly in young adolescents, is yet to be fully understood (Leone & Wade, 2018). Little is known sex differences across perfectionism and what implications this may have for interventions (Smith et al., 2021). Only two studies have evidenced differences between profiles of perfectionism between males and females in latent cluster analyses (Sironic & Reeve, 2015; Stornæs et al., 2019), which warrants further exploration in this area.

To date, universal prevention programs for perfectionism delivered using external facilitators have yielded promising results (Fairweather-Schmidt & Wade, 2015; Nehmy & Wade, 2015; Vekas & Wade, 2017; Wilksch et al., 2008) and show significant decreases (*d* = 0.35–1.34) in perfectionism over 4-week to 12-month follow-up compared to control conditions, along with decreases in negative affect (*d* = 0.27) and improvement in well-being (*d* = 0.33). Interventions such as Vekas & Wade (2018) have placed emphasis on the difference in pursuing high standards versus perfectionistic strivings, and the usefulness in practicing self-compassion as a way of encouraging perseverance in the face of difficulties (Gilbert, 2014).

The main aim of the current research was to examine the efficacy of a 5 lesson intervention in young adolescents delivered by classroom teachers. The development of school-based programs with teachers as facilitators has been proposed as an advantageous way to disseminate programs more widely (Han & Weiss, 2005). However, to date no research has examined the efficacy of this modality for the implementation of a perfectionism program. To this end we utilized measures of our primary outcomes, perfectionism, and high standards, using the Frost Multidimensional Perfectionism Scale (FMPS; Frost et al., 1990), the Almost Perfect Scale-Revised (APS-R; Slaney et al., 2001), and Hewitt Multidimensional Perfectionism Scale (HMPS; Hewitt & Flett, 1991) to examine efficacy of the intervention. Subscales reflecting perfectionistic strivings included a combination of Personal Standards (FMPS) and Self-Oriented Perfectionism (HMPS) as per a priori factor-analytic evidence (Cox et al., 2002), while the High Standards subscale (APS-R) was considered as a measure of pursuit of high standards (Blasberg et al., 2016). Perfectionistic concerns were assessed exclusively using the Discrepancy subscale (APS-R), as it has been shown to be one of the more harmful measures of perfectionistic concerns (Limburg et al., 2017; Osenk et al., 2020). Following recommendations from previous literature (Stoeber and Otto, 2006), we focused outcomes solely on individually oriented perfectionism in young adolescents, which is the core target of clinical interventions (Shafran et al., 2002). Thus, FMPS-organization, HMPS-other-oriented perfectionism, and APS-R-Order were excluded from this review. FMPS-parental expectations and parental criticism were omitted as evidence suggests these are best understood as preceding factors involved in upbringing (Stoeber & Otto, 2006). Our secondary outcomes of interest included well-being, negative affect, self-compassion, and academic intrinsic motivation.

We hypothesized that, compared to a control group, the intervention would result in decreased levels of perfectionistic concerns and perfectionistic strivings, but not high standards, as well as higher levels of well-being, intrinsic motivation, and lower levels of negative affect. Given samples with elevated levels of psychological distress display greater benefits from interventions (Werner-Seidler et al., 2017), we also investigated whether those with higher levels of perfectionistic concerns, and thus at greater risk of maladaptive outcomes (Limburg et al., 2017), would have greater benefit from the intervention. Finally, we also investigated whether sex would moderate the impact of the intervention given the little yet well-needed exploration in this area.

## Method

### Participants

Six hundred and thirty-six high school participants (*M*_age_ = 13.68, *SD* = 0.60; 52.8% female) across Year 8 and Year 9 agreed to take part in the current study. A range of co-education and single-sex secondary schools in Adelaide, South Australia, were contacted by e-mail and telephone, and four schools (three co-educational, one female-only) consented their participation. These schools reflected a high socio-economic status on the Index of Community Socio-Educational Advantage (ICSEA; Australian Curriculum Assessment & Reporting Authority, 2012), whereby 1000 represents the mean, with a standard deviation of 100. The schools ranked in the top 20 schools in South Australia and ranged from 1122–1173, with a mean index of 1150 (*SD* = 21.30). Two schools were recruited and completed the program in 2019 prior to the COVID-19 pandemic (*n* = 447), and two schools completed the program within the following year in 2020 during the COVID-19 pandemic (*n* = 189). There was no change in protocol, including intervention implementation and data collections that occurred due to the COVID-19 pandemic. A power analysis was conducted for repeated measures designs (Hedeker et al., 1999). Three time points were included and the analyses included a planned effect size of 0.30 with an alpha level of 0.05, two sided test, and a power level of 0.80. The analyses required N = 103 entered for each group (N = 206 students in total). The effect size was chosen because of previous evaluation of school intervention in perfectionism (Nehmy & Wade, 2015) showed a 0.24 between group difference at 12 month follow-up.

### Design

Classes were randomized to the perfectionism lessons or wait-list control condition by the first author, using the randomization function in Excel 2016.

#### Procedure

Approval for the research was granted by the Flinders University Social and Behavioral Research Ethics Committee (Project Number 7901) and the South Australian Department for Education and Child Development (Application 2018–0003). Informed consent was obtained by the principal of the participating school, and from each participating student (assent) together with passive consent from their parent or guardian. All students in the intervention condition completed the program as it was embedded in their curriculum, but consent was obtained for the completion and use of questionnaire data in research. Students in the control condition received the intervention after the 3-month follow-up period was completed. All participants filled out questionnaires online on their personal laptop devices using Qualtrics Survey software. Testing was performed in a classroom setting, with students requested to comply with standard test conditions (i.e., working silently and independently), with either the first author, a research assistant holding a degree in Psychology, or a teacher, available to answer any questions. The intervention lessons were delivered by their regular class teacher following a 2 h workshop delivered by the first author.

### Measures

Participants completed the following measures at three time points (baseline, post-intervention and 3-month follow-up) and mean item total scores used, where higher scores indicate higher levels of the construct in question.

#### Perfectionism

The High Standards (7 items) and Discrepancy (11 items) subscales from the Almost Perfect Scale-Revised (APS-R; Slaney et al., 2001) were utilized in the current study. Items are rated on a 7 point scale ranging from 1 (*strongly disagree*) to 7 (*strongly agree*). High Standards is intended to assess striving for high standards of performance while Discrepancy assesses the perception that personal high standards are not being met. Previous research in children has reported construct validity, and good to very good internal consistency for the subscales (*α* = 0.79–0.89) and confirmed the distinction between the subscales (Leone & Wade, 2018). Due to limitations with the assumptions underlying Cronbach’s alpha (McNeish, 2018), Coefficient H was computed as an indicator of internal reliability at baseline, giving values of 0.88 and 0.92 respectively, which can be interpreted in a similar fashion to Cronbach’s alpha.

The Frost Multidimensional Perfectionism Scale (FMPS; Frost et al., 1990) is a 35 item questionnaire of which only the 7 item Personal Standards subscale was utilized e.g., “It is important to me that I am thoroughly competent in everything I do”. Respondents are asked to rate their response on a 5 point scale ranging from 1 (*strongly disagree*) to 5 (*strongly agree*). Previous research has established sound psychometric properties for the use of the FMPS in children and adolescents, with Gavino et al., (2019) demonstrating internal consistency between α = 0.71 and 0.92, test–retest reliability with intraclass correlations ranging between 0.70 and 0.85. In the current study baseline coefficient H was 0.86.

The Hewitt Multidimensional Perfectionism Scale comprises of 45 items (HMPS; Hewitt & Flett, 1991), of which only the 5-item self-oriented perfectionism subscale from the short-form of this questionnaire were utilized due to the advantages of its shortened length and ease of administration with youth in mind (Nealis et al., 2020; Smith et al., 2015), e.g., “I demand nothing less than perfection of myself”. Items are rated on a 7-point scale ranging from 1 (*strongly disagree*) to 7 (*strongly agree*). Previous research has established a respectable relationship between the short form of the HMPS and the original questionnaire (*r* = 0.81-0.90; Hewitt et al., 2008), the same relationship with various outcomes as the original questionnaire. The original HMPS demonstrates good internal consistency, test–retest reliability, and construct validity (Hewitt & Flett, 2004). Baseline coefficient H was 0.90.

As per previous studies (Cox et al., 2002; Stoeber & Otto, 2006), the Personal Standards and Self-Oriented Perfectionism subscale were standardized into *z* scores and then averaged together to create a perfectionistic strivings composite score for main analyses.

#### Well-being

The Warwick-Edinburgh Mental Well-being Scale (WEMWBS) is a 14 item scale (Tennant et al., 2007) and is considered suitable to measure mental wellbeing in youth (Clarke et al., 2011). Participants are asked to circle the number that best describes their experience of a statement over the past two weeks using a 5 point Likert scale, from 1 “*none of the time”* to 5 *“all of the time”*. The WEMWBS has demonstrated sound psychometric properties in populations of young adolescents from 13 years and over including internal consistency (α = 0.82–0.87), test–retest reliability, and construct validity (Clarke et al., 2011; Tennant et al., 2007). At baseline Coefficient H was 0.92.

#### Self-compassion

The 26-item, six-subscale self-compassion scale (SCS: Neff, 2003) has been used across many different studies with a 12-item short form (SCS-SF; Raes et al., 2011) developed for adults across three samples with mean ages of 18.14, 33.04 and 20.62, respectively. For the short form, two items from each subscale of the SCS are included based on (i) high correlations with the long SCS scale, (ii) high correlations with the intended SCS subscale, and (iii) high correlations between the two items that accounted for the breadth of the original subscale. The correlation between the long- and short-form total scores was near perfect (*r* = 0.97). Participants provide self-report ratings on a 5-point Likert scale, from 1 “*almost never*” to 5 “*almost always*”, and all items were coded for this study such that higher scores indicated higher levels of self-compassion. In the current study the baseline Coefficient H was 0.81.

#### Negative Affect

The 14 items from the depression and anxiety subscales from the 21-item Depression, Anxiety, and Stress Scale (DASS-21; Lovibond & Lovibond, 1995) were used to assess negative affect. For each item, participants are asked to rate how much each statement applied to them over the past week on a four-point scale from 0 “*did not apply to me at all*” to 3 “*applied to me very much, or most of the time*”. The DASS-21 has been psychometrically validated in a large sample of South Australian adolescents (Tully et al., 2009). At baseline Coefficient H was 0.93.

#### Academic Motivation

The 28 item Academic Motivation Scale (AMS; Vallerand et al., 1992) comprises seven subscales of which the three intrinsic motivation subscales (orientation toward stimulating experiences, orientation toward achievement, orientation toward knowledge) were used to assess engagement in academic activities for intrinsic reasons. Respondents were asked to rate their agreement with each item on a 7 point scale ranging from 1 “*Does not correspond at all*” to 7 “*Corresponds exactly*”. The AMS has been used extensively in adolescent populations and demonstrated sound psychometric reliability and validity (Utvær & Haugan, 2016) and in our study coefficient H was 0.95.

### Pilot Intervention

A three-lesson version of the program has been described previously in children aged 10–12 (Vekas & Wade, 2017), informed by the cognitive behavioral model of clinical perfectionism (Shafran et al., 2002). It was pilot tested with lessons delivered one week apart to gifted year 8 students (*N* = 93; 39 females; *M*^age^ = 13.59, *SD* = 0.40), delivered by the first and second authors. Classes were randomized to the intervention or a lesson-as-usual control condition. Assessments were completed at baseline, post-intervention, and three-month follow-up. Data were analyzed using linear mixed models with both baseline observation and age included as covariates. At post-intervention small between-group effect sizes (Cohen’s *d*) were obtained for discrepancy (0.40: 95% confidence intervals [CI]: − 0.02:0.81), self-compassion (0.36: − 0.05:0.77) and negative affect (0.20: − 0.21:0.61), favoring the intervention group, but the commensurate effect size for high standards was negligible (0.07: − 0.34:0.48). At 3-month follow-up, self-compassion retained a small between-group effect size favoring the intervention group (0.30: − 0.11:0.71).

## Current Intervention

To increase effect sizes obtained in the pilot study, the 3-lesson program was modified to a 5-lesson program to boost the observed helpful effects, increase the module on self-compassion and self-criticism and to include a social media component (see Table 1). Teachers delivered the intervention via a structured protocol and were required to complete a checklist to ensure uniformity in lesson delivery, with 100% compliance rate. All lessons were each delivered a week apart, were 45 min in length, and content was delivered in an engaging and interactive manner as opposed to a didactic presentation, as the former has been shown to produce larger effect sizes (Stice et al., 2007). This included use of brainstorming activities, small group and whole class discussions, out-of-class experiments, and individual-orientated reflective exercises. Control students completed assessment only and participated in their usual class lessons.

**Table 1:** Lesson Outline for the 5-lesson perfectionism program

Theme	Outline of lesson
1. Unhealthy perfectionism versus the pursuit of excellence	Class discussion: What is the difference between perfectionism and the pursuit of excellence? How does trying to be perfect feel – and what are the pitfalls?
	JK Rowling’s 10 important lessons for success (video/small group discussion)
	Failure helps you discover yourself; Take action on your ideas; You will be criticized; Remember where you started; Believe; There is always trepidation; Life is not a checklist of achievements; Persevere; Dreams can happen; We have the power to imagine better
	Take home activity: Choose one of the tips and illustrate as a poster
2. Three tips to be a successful learner	Sharing of posters
	Taking time out will improve your performance
	Yerkes Dodson Law – class activity to discuss the quote “the harder you study the better you perform—true or false”
	Making mistakes and failing is an essential part of success
	Small group discussion: What are the advantages of making mistakes?
	Celebrating Success is Good for You
	Write down 5 things you enjoy doing – just for you
	Home activity experiment: Experiment with taking time out, getting 8 h of sleep, and incorporating 5 things you enjoy doing. Assess productivity with study
3. The power of self-compassion	Home activity recap
	How to react when things don’t do as well as you had hoped or planned
	Recall and discuss such a situation in small groups – what were your thoughts, feelings and behaviors?
	Class discussion: What is self-compassion? What does it look like? (thoughts/feelings/behaviors)
	e.g., being kind to yourself in the face of failure, getting ‘back on the horse’, allowing yourself room to accept mistakes
	In class activity: Writing a compassionate letter to a friend after a disappointment
	Take home activity: Writing a compassionate letter to self after a disappointment and rate mood before and after
4. Self-Compassion versus self-criticism	Home activity recap
	Story: Coach Curly (critical) vs Coach Moe (encouraging)
	Which coach would you choose for your friends and why? Which coach would produce a better performance?
	The tripod of balance
	•Threat, Achievement and Compassion
	•Class discussion: What happens if one is missing?
	Class activity: Role Play of Mr. Compassionate versus Mr Critical
	Practicing the generation of self-compassionate thoughts in response to self-critical comments after a scenario of failure
	Small group brainstorm: what are some things we can do when we feel critical of ourselves?
	e.g., Keep a self-compassion journal, write yourself a letter (last week’s activity), Go for a walk or talk to a friend/family member
	Take home activity: CBT self-compassionate thought log
5. Social Media and Perfection	Report back on home activity
	Small group activity: what influence do you think social media has on trying to be perfect?
	Living up to impossible standards, everybody posts their perfect selves on social media – feelings of sadness, frustration when not living the “perfect” live compared to others, pressure to get likes and comments on photos to look popular
	Video: Social Media and Perfection (in class discussion)
	What are the main messages of the video? Does your real life differ from your online life? How is it different? Why? How can this’perfect ideal’ on social media be problematic?
	Small Group Activity: Reflection on program
	What did you find most helpful and why?
	Take home activity: take a photo of something ‘real’ that happened to you during the week (i.e., something you would never post online on social media) and place on social media template to hang in class as a reminder that life isn’t perfect

## Statistical Analyses

### Repeated Measures Analyses

Linear Mixed Models (LMM) accounts for correlations and non-independence among observations and allows for an intent-to-treat analysis as it accommodates missing data by using Restricted Maximum Likelihood (REML). It assumes data are missing at random (MAR; Han & Guo, 2014). To identify whether there were baseline predictors of missing data at post-intervention or 3-month follow up, logistic regressions were conducted.

In the LMM, an unstructured covariance matrix was assumed, and models were adjusted for the effect of clustering, given that different teachers delivered the perfectionism classes. All analyses were adjusted for baseline observations to ensure that outcomes resulted from intervention-related influences and not measurement error or baseline score differences, and therefore significant between-group and interaction terms were both of interest. This resulted in a 2 (group: intervention, wait-list control) × 2 (time: post-intervention, 3-month follow-up) repeated measures design. Bonferroni corrections were applied to all post hoc comparisons. Between-group Cohen’s *d* effect sizes were calculated, where 0.2 = small; 0.5 = moderate, and 0.8 = large (Cohen, 1992). All models were also examined for three-way interactions with the following variables: sex and “maladaptive” perfectionistic concerns e.g., Discrepancy, defined by Rice and colleagues (2011) as a mean item total score ≥ 3.5. This resulted in a 2 (group: intervention, wait-list control) × 2 (time: post-intervention, 3 month follow-up) × 2 (moderator) repeated measures design.

## Results

### Participant Retention and Baseline Comparisons

**Fig. 1:**
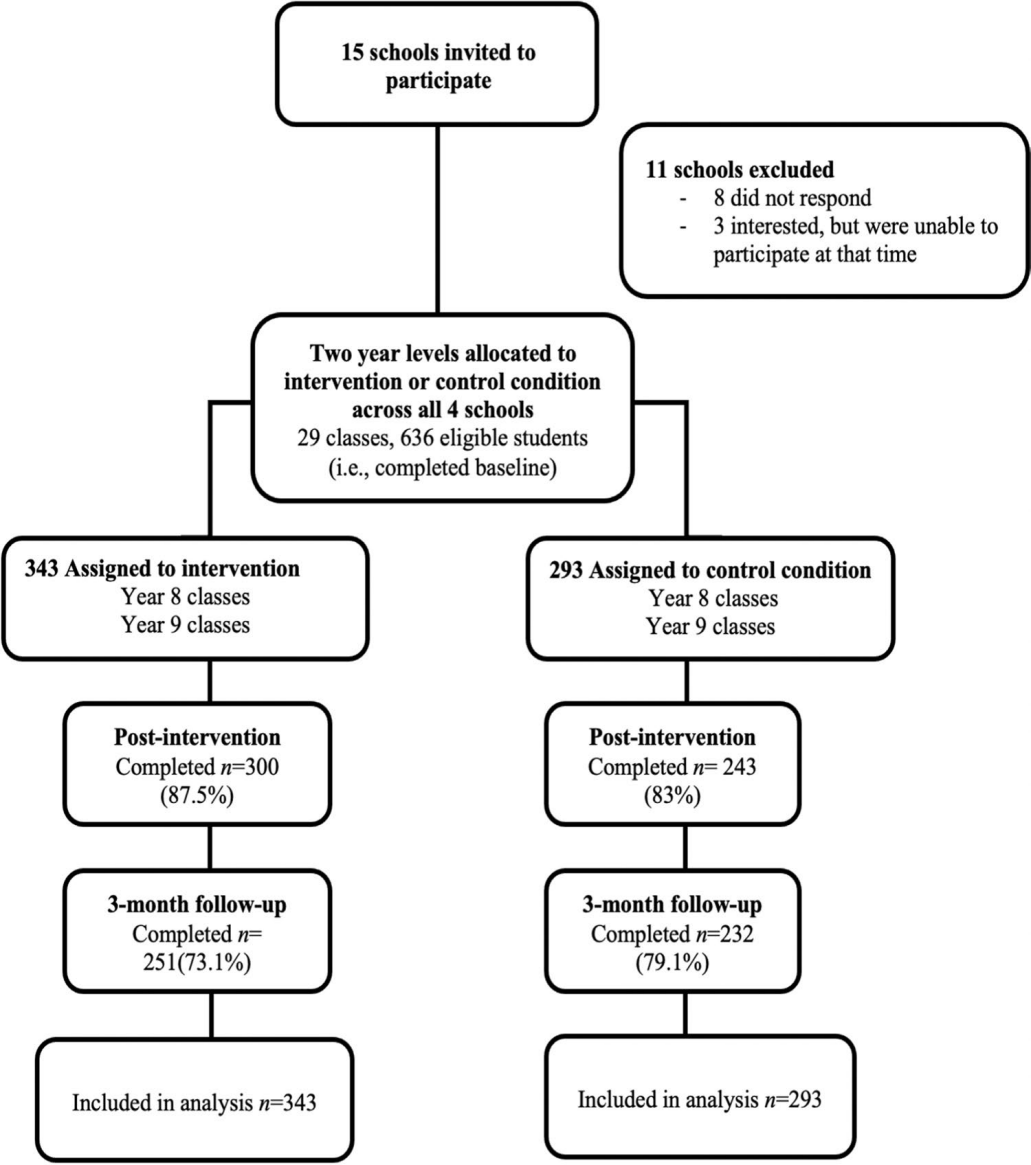
Flow of participants through the intervention

Figure 1 presents the recruitment and retention of participants in each group over the three waves of data collection (baseline, post-intervention, 3-month follow-up), with missing data for 15% and 24% at post-intervention and 3-month follow-up, respectively. Baseline comparisons of those who had complete data across all three time points and those who did not, reported in Table 1, showed data to be missing at random. There were no significant differences between treatment groups at baseline assessment (Table 2) and no significant differences between schools who participated during COVID-19 and those who did not, except for sex due to the recruitment of an all girls school during the COVID period.

**Table 2:** Investigation of missing at random and any group differences at baseline using logistic regression

Variable	Comparing those with missing and complete data at each wave				Comparisons between groups			
	Post-intervention OR (95%CI)	3-month follow-up OR^a^ (95% CI)	Control (*N* = 293) (*M, SE*)	Intervention (*N* = 343) (*M, SE*)	OR (95%CI)	COVID (*N* = 189) (*M, SE*)	Non-COVID (*N* = 447) (*M, SE*)	OR (95% CI)
High Standards	0.95 (0.53, 1.70)	1.08 (0.67, 1.76)	5.54 (.06)	5.29 (0.06)	1.15 (0.82, 1.63)	5.45 (0.09)	5.39 (0.05)	1.09 (0.62, 1.92)
Discrepancy	0.91 (0.56, 1.48)	1.26 (0.82, 1.93)	3.91 (0.08)	3.89 (0.07)	0.95 (0.72, 1.26)	3.85 (0.10)	3.93 (0.06)	0.85 (0.54, 1.36)
Personal Standards	1.28 (0.51, 2.34)	0.76 (0.35, 1.66)	3.10 (0.05)	2.98 (0.05)	1.10 (0.65, 1.86)	3.34 (0.06)	2.88 (0.04)	0.94 (0.43, 2.03)
Self-Oriented Perfectionism	0.82 (0.52, 1.28)	1.09 (0.75, 1.57)	3.72 (0.14)	3.79 (0.13)	0.90 (0.70, 1.15)	3.85 (0.11)	3.58 (0.16)	1.28 (0.91, 1.81)
Depression	1.28 (0.12, 14.21)	0.32 (0.10, 1.00)	0.61 (0.03)	0.64 (0.03)	0.98 (0.43, 2.24)	0.68 (0.05)	0.60 (0.02)	1.39 (0.39, 4.95)
Anxiety	8.85 (0.81, 96.89)	0.41 (0.13, 1.33)	0.61 (0.03)	0.63 (0.03)	0.81 (0.37, 1.80)	0.70 (0.04)	0.58 (0.03)	1.38 (0.40, 4.83)
Wellbeing	1.06 (0.45, 5.26)	0.59 (0.59, 2.93)	3.58 (0.04)	3.57 (0.04)	0.83 (0.46, 1.50)	3.52 (0.06)	3.60 (0.03)	1.83 (0.74, 1.53)
Self-compassion	0.31 (0.10, 0.96)	0.75 (0.31, 1.83)	3.97 (0.04)	3.98 (0.03)	0.95 (0.52, 1.71)	3.90 (0.05)	4.00 (0.03)	0.77 (0.29, 2.04)
Intrinsic Motivation	0.93 (0.54, 1.58)	0.80 (0.50, 1.29)	4.90 (0.07)	4.67 (0.07)	1.14 (0.83, 1.58)	4.67 (0.10)	4.83 (0.06)	1.02 (0.61, 1.69)
Sex	0.47 (0.18, 1.21)	0.53 (0.23, 1.20)	147 female; 50%	189 female; 55%	1.02 (0.57, 1.84)	118 female; 63%	218 female; 47%	0.09 (0.03, 0.26)*

OR = odds ratio. CI = confidence intervals. ^a^ 3-month follow-up predicted from baseline. * = significant at *p* < .001

### Repeated Measures Analyses

All variables met assumptions for normality. Table 3 reports estimated marginal means for main group effect, and effects of time and interaction with time and group effects. One interaction between time and group was found for anxiety whereby small effect size changes were found, favoring the intervention group. Anxiety showed an increase in the control group while appearing stable in the intervention group, resulting in a non-significant between-group effect size difference of *d* = 0.07 (95% CI: − 0.11, 0.23) at post-intervention and significant between-group effect size difference of *d* = 0.23 (95% CI: 0.05, 0.40) at 3 month follow up, respectively.

**Table 3:** Adjusted means and standard errors across time and main effects and interactions

Variable	Baseline Covariate	Post-intervention	3-month Follow-Up	Time	Condition	Time x Condition
	*M*	*M* (*SE*)	*M* (*SE*)			
Perfectionistic Concerns						
Intervention	3.94	3.84 (0.06)	3.83 (0.07)	*F*(1, 449.80) = 0.01 *p* = .95	*F*(1, 23.04) = 1.67 *p* = .21	*F*(1, 449.97) = 0.06 *p* = .81
Control		3.94 (0.07)	3.95 (0.07)			
High Standards						
Intervention	5.45	5.27 (0.06)	5.14 (0.06)	***F*(1, 473.20) = 10.78 *p* = .001**	*F*(1, 26.69) = 0.58 *p* = .45	*F*(1, 473.37) = 0.22 *p* = .64
Control		5.34 (0.06)	5.18 (0.07)			
Perfectionistic Strivings^a^						
Intervention	− 0.13	− 0.23 (0.04)	− 0.05 (0.04)	*F*(1, 467.33) = 2.55 *p* = .11	*F*(1, 35.57) = 0.01 *p* = .94	*F*(1, 473.37) = 0.22 *p* = .54
Control		− 0.01 (0.04)	− 0.07 (0.05)			
Depression						
Intervention	0.63	0.64 (0.02)	0.698(0.03)	***F*(1, 461.38) = 10.47 *p* = .001**	*F*(1, 25.60) = 0.00 *p* = .99	*F*(1, 461.34) = 1.74 *p* = .19
Control		0.60 (0.03)	0.72 (0.03)			
Anxiety						
Intervention	0.63	0.64 (0.02)	0.62 (0.02)	*F*(1, 469.87) = 1.82 *p* = .28	*F*(1, 27.70) = 0.76 *p* = .39	***F*(1, 469.79) = 5.38 *p* = .02**
Control		0.62 (0.03)	0.69 (0.03)			
Well-being						
Intervention	5.57	3.56 (0.03)	3.53 (0.03)	***F*(1, 466.04) = 4.15 *p* = .04**	*F*(1, 27.73) = 0.11 *p* = .74	*F*(1, 465.98) = 0.79 *p* = .37
Control		3.60 (0.03)	3.52 (0.04)			
Self-compassion						
Intervention	3.96	4.00 (0.03)	3.99 (0.03)	*F*(1, 471.07) = 2.58 *p* = .11	*F*(1, 563.60) = 1.64 *p* = .20	*F*(1, 471.16) = 1.10 *p* = .29
Control		3.98 (0.03)	3.91 (0.03)			
Intrinsic motivation						
Intervention	4.81	4.69 (0.06)	4.61 (0.07)	***F*(1, 461.63) = 4.42 *p* = .04**	*F*(1, 28.75) = 0.75) *p* = .39	*F*(1, 461.55) = 0.10 *p* = .75
Control		4.78 (0.07)	4.66 (0.07)			

Higher scores indicate poorer outcomes for depression, anxiety, better outcomes for well-being, self-compassion, intrinsic motivation and extrinsic motivation and greater perfectionism on all perfectionism measures. Significant effects are bolded. ^a^ mean and standard error reported in *z* score format

No other between-group differences were found. There was no evidence to suggest that the lessons impacted on any perfectionism variable, including high standards, perfectionistic concerns, or perfectionistic strivings at either post-intervention or 3 month follow-up, with between-group effect sizes ranging between 0.02 and 0.18.

Main effects of time were observed for high standards, perfectionistic strivings, well-being, depression, and intrinsic motivation whereby levels of perfectionistic strivings and depression significantly increased over post-intervention and 3 month follow-up, while levels of high standards, well-being and motivation significantly decreased over follow-up.

## Moderation Analyses

**Fig. 2:**
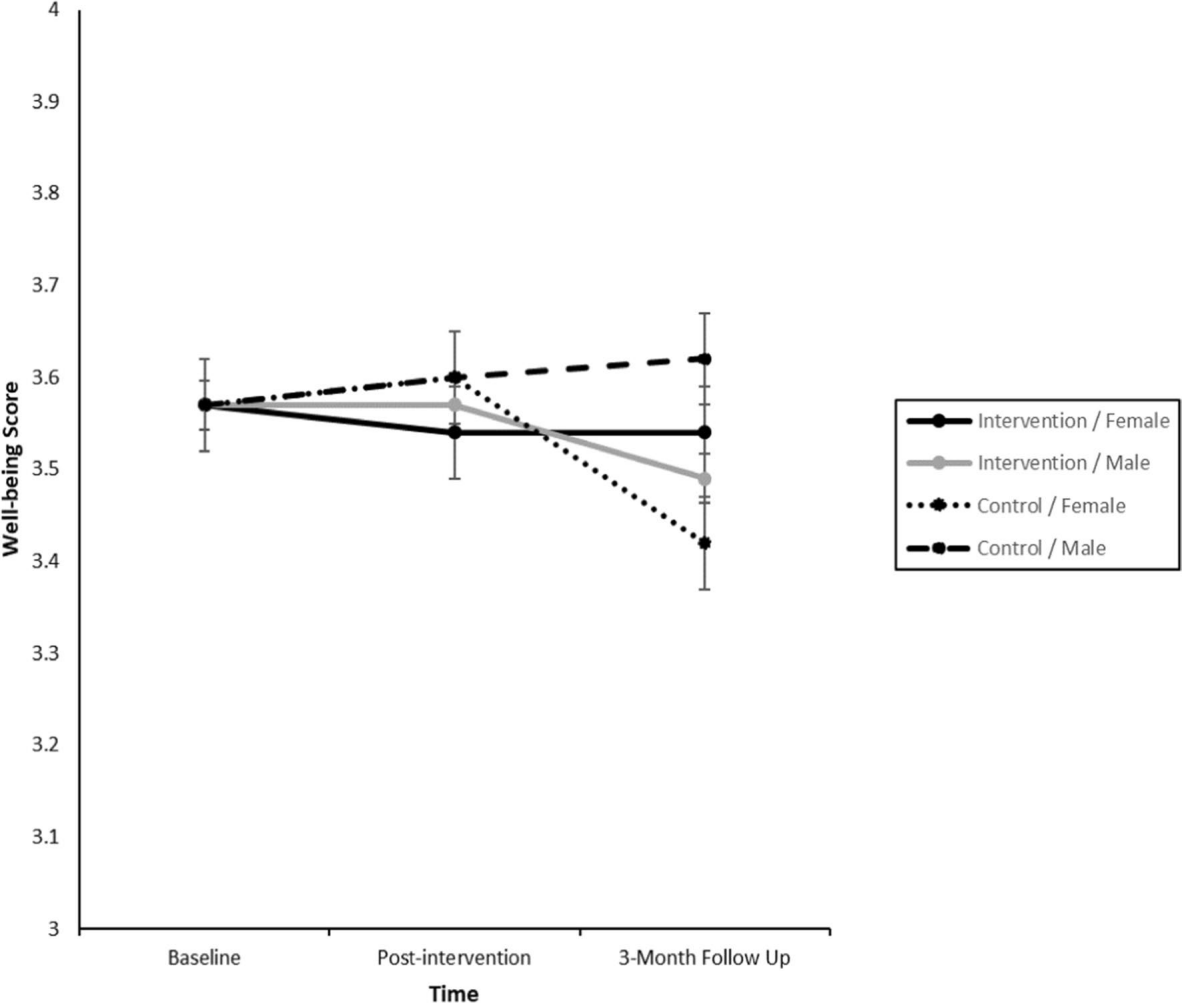
Changes to Well-Being across Time by Group (Intervention, Control) x Sex (Male, Female) Analysis was adjusted for baseline observations: covariate value for well-being was 3.57

Across a series of three-way interactions examining the relationship with sex and problematic perfectionism, two significant three-way interactions were found involving sex (see *Supplementary Table 1* and Fig. 2). Well-being remained stable in the intervention group across both males and females, but significantly decreased for females only in the control group between post-intervention and 3 month follow-up, with a between-group effect size of *d* = 0.33 (95% CI: 0.08, 0.58) at 3 months. Intrinsic motivation appeared stable for females in the intervention group, and for males in the control group, but decreased between post-intervention and 3-month follow-up for males in the intervention group, and females in the control group. Post-hoc analyses for intrinsic motivation revealed no significant differences between males and females in the intervention and control group at any timepoint.

**Fig. 3:**
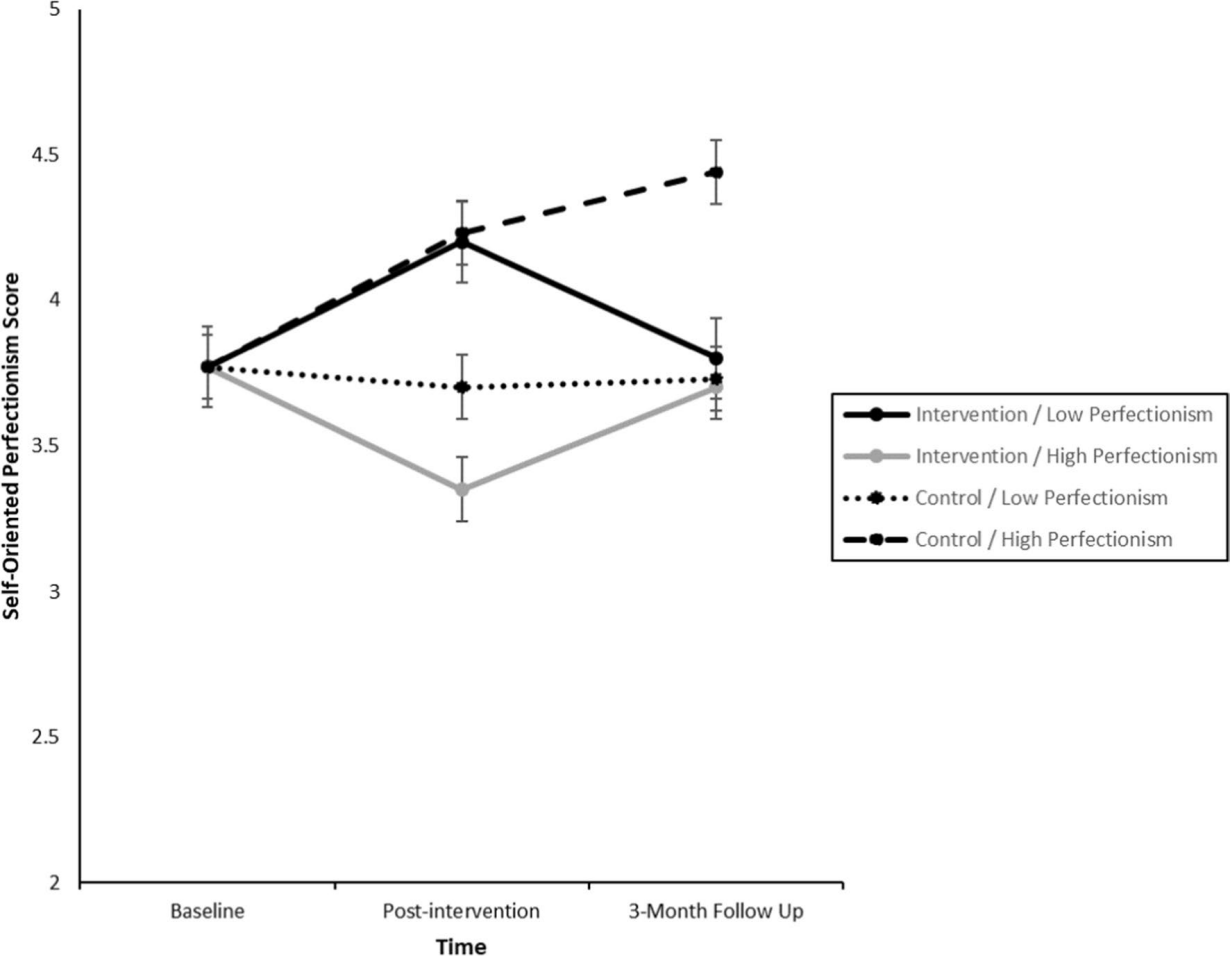
Changes to Perfectionistic Strivings across Time by Group (Intervention, Control) x Perfectionistic Concerns Level (Low, High) Analysis was adjusted for baseline observations: covariate value for perfectionistic strivings was 3

*Supplementary Table 2* and Fig. 3 presents results for the three-way interactions involving perfectionistic concerns at a “maladaptive” level. Those in the intervention group with a maladaptive level of perfectionistic concerns experienced decreased levels of perfectionistic strivings while those below the cut-off experienced an increase in perfectionistic strivings, with significant between-group effect size of *d* = 0.40 (95% CI: 0.25, 0.56) at post-intervention, and both returning to baseline levels at 3 month follow-up with no difference between the two groups. For those in the control group, students with higher levels of “maladaptive” perfectionistic concerns had a significant increase in perfectionistic strivings at post-intervention and 3-month follow-up compared to those below the cut-off, *d* = 0.27 (95% CI: 0.11, 0.44) and *d* = 0.36 (95% CI: 0.20, 0.53), respectively. Of most interest are those students with higher levels of “maladaptive” perfectionistic concerns; the intervention group achieved a significantly lower level of perfectionistic concerns at 3-month follow-up (*d* = 0.34, 95% CI: 0.19, 0.49) than the control group.

## Discussion

The present study represents the third evaluation of a universal perfectionism intervention (Fairweather-Schmidt & Wade, 2015; Vekas & Wade, 2017), and the first evaluation of a teacher-led perfectionism program. Unlike the previous two evaluations, the present study did not yield any significant decreases in either perfectionistic strivings or concerns. These findings were somewhat surprising given our previous evaluations, which demonstrated the efficacy of decreasing perfectionistic strivings (e.g., *d* = 0.47–0.40, Fairweather-Schmidt & Wade, 2015; *d* = 0.35, Vekas & Wade, 2017). Our findings were also not accompanied by convincing changes in depression, self-compassion, well-being in boys, or academic motivation. Rather, significant effects of time were observed, indicating increases in depression and perfectionistic strivings, and decreases in well-being, high standards and academic motivation across both groups. These findings reinforce the critical need for the development of universal school-based programs for young adolescents, whom are at high risk of developing mental health difficulties (Sawyer et al., 2018).

However, the intervention was successful in preventing the increase in anxiety over 3 month follow-up compared to the control group (*d* = 0.23). This is an important finding given that school-based intervention programs for psychological distress tend to report non-significant findings for adolescents (Ahlen et al., 2015) and only three targeting anxiety have shown significant effects of *d* = 0.22–0.70 at follow-up (Neil & Christensen, 2009). Considering the current research, the prevention of anxiety elevation throughout the school year using a teacher-led program is significant when considering the detrimental effects that anxiety has in youth well-being and education (Bittner et al., 2007; Pine et al., 1998). However, we cannot attribute the mechanism of this result to a decrease in perfectionism and investigating the mechanisms by which the intervention elicited this effect should be examined in further research.

The lack of significant findings thus raises questions about the optimal delivery mode for perfectionism prevention in young adolescents. While we cannot directly compare teacher-led vs. psychologist-led results, our results appear to suggest that psychologist-led programs may be helpful for this population. Indeed, there is evidence to suggest that prevention programs delivered by external facilitators such as psychologists may be more efficacious in nature compared to those delivered by teachers (Stice et al., 2009; Wahl et al., 2014). To the best of our knowledge, our study was the first to examine the use of a universal school-based intervention program facilitated by teachers in the field of perfectionism, and future research should endeavor to directly compare the effectiveness of this intervention when led by trained professionals vs. teachers.

Our findings may also indicate that the students’ environment in terms of peer, teacher, school and/or parent pressures around achievement may play a role in the impact of the intervention. Indeed, the external environment has been posited to play a pivotal role for the development of perfectionism in adolescence (Flett et al., 2002). Evidence suggests perceived pressure from parents and teachers significantly increases the incidence of perfectionistic concerns, while receiving perceived teacher support decreases perfectionism (Domocus & Damian, 2018). Interventions from other disciplines such as sleep have reported great benefit from the inclusion of parents in intervention work, such as the provision of education (Bonnar et al., 2015). Taken together with the detrimental effects of perceived expectations of performance on perfectionism, it may be that parental involvement and inclusion is an important area of research to consider in future work.

Consistent with evidence that interventions targeting high risk children yield significantly better results (Werner-Seidler et al., 2017), we did find that for students with high levels of perfectionistic concerns, those who received the intervention had significantly lower levels of perfectionistic strivings at 3-month follow-up than those in the control condition. Thus, the intervention shows promise with curtailing a naturally occurring growth of perfectionistic strivings in youth displaying high levels of perfectionistic concerns, who are at an increased risk of psychopathology and impairments to learning (Hewitt et al., 2002). Our results are also consistent with meta-analytic findings that have found no differences in benefits of intervention programs based on sex for other intervention programs (i.e., Ahlen et al., 2015; van Loon et al., 2020). However, females who participated in the intervention group were protected from significant deterioration in well-being compared to females in the control group. This finding is promising given epidemiological and clinical studies have generally found higher incidences and increased risk of psychopathology in females compared to males (Eaton et al., 2012; Klose & Jacobi, 2004). These findings illustrate the particular importance of universal-based prevention programs for females in preventing deterioration of mental health.

The results should be interpreted in the context of the following limitations. First, random sampling of the general population was not achieved, including omitting groups with lower SES, which is a significant predictor of mental health difficulties in adolescents (Reiss, 2013). Future research should endeavor to implement such universal-based programs with greater demographic information and examine SES status as a moderator of intervention benefit. Second, while fidelity of program implementation was formally assessed via a checklist and data analysis accounted for classroom membership, observations of teacher facilitation were not conducted. Future research focusing on teacher-led interventions should measure program implementation to inform generalizability of program dissemination. Third, longer follow-up is required given sleeper effects for post-intervention differences in universal prevention, where the impact of prevention programs may only become apparent over time as psychopathology increases (Nehmy & Wade, 2015). Finally, it would be more appropriate for future research to also include the child and adolescent version of the HMPS, the CAPS (Flett et al., 2016). It should be noted the construct validity of perfectionism measures in young adolescents is generally poor (Leone & Wade, 2018). This broader issue on the validity of perfectionism measures should be placed as a priority for future research to glean clarity on the way we measure perfectionism in youth. Similarly, the SCS has not been formally validated with young adolescents. A Self-compassion Scale for Youth has recently been validated for use in children and young adolescents (Neff et al., 2020), and future research should endeavor to utilize this measure when examining self-compassion in these populations.

In summary, given the rise in perfectionism in youth (Curran & Hill, 2019), and the range of unhelpful consequences for both mental health and academic achievement (Gilman et al., 2010; Limburg et al., 2017) the development of effective interventions for decreasing perfectionism while retaining high standards, is imperative. The current study suggests promising findings in preventing elevated levels of anxiety over time, curtailing increases in perfectionistic strivings in youth with high levels of perfectionistic concerns, and specifically protect well-being levels in females. More research is required to replicate these findings in larger and more diverse samples, and further development of this program is encouraged with respect to length, content and exploring the optimal facilitator of the program.

## Supplementary Information

Below is the link to the electronic supplementary material.

Supplementary file1 (DOCX 25 kb)

## Data Availability

The data that support the findings of this study are available from the corresponding author upon reasonable request.
