# Supplementary material for: A Pragmatic Randomized Controlled Trial Targeting Perfectionism in Young Adolescents
Source: School Ment Health. 2022 Sep 6;15(1):151–64. doi: 10.1007/s12310-022-09540-3 (PMC9446653; doi:10.1007/s12310-022-09540-3)
Supplement: Supplementary file 1 — Supplementary file1 (DOCX 25 kb) [file 12310_2022_9540_MOESM1_ESM.docx]

Supplementary Table 1

*Adjusted Means and Standard Errors across Time and Interactions with Condition, Time and Sex.*

| Outcome measure | Baseline covariate  (*M*) | Post-intervention  *M* (*SE*) | 3-month follow-up  *M* (*SE*) | Condition x time x sex |
| --- | --- | --- | --- | --- |
| *High Standards* |  |  |  |  |
| Intervention | 5.45 |  |  |  |
| Male |  | 5.20 (0.08) | 4.94 (0.10) | *F*(1, 474.65) = 1.82*, p* = .181 |
| Female |  | 5.29 (0.07) | 5.24 (0.08) |  |
| Control |  |  |  |  |
| Male |  | 5.24 (0.09) | 5.09 (0.09) |  |
| Female |  | 5.43 (0.08) | 5.25 (0.09) |  |
| *Perfectionistic Concerns* |  |  |  |  |
| Intervention | 3.93 |  |  | *F*(1, 449.58) = 0.09*, p* = .762 |
| Male |  | 3.87 (0.10) | 3.79 (0.11) |  |
| Female |  | 3.80 (0.08) | 3.81 (0.09) |  |
| Control |  |  |  |  |
| Male |  | 3.89 (0.10) | 3.89 (0.10) |  |
| Female |  | 3.96 (0.10) | 3.99 (0.10) |  |
| *Perfectionistic Strivings* |  |  |  |  |
| Intervention | 3.77 |  |  | *F*(1, 235.01) = 0.14*, p* = .711 |
| Male |  | 3.62 (0.17) | 3.73 (0.16) |  |
| Female |  | 3.53 (0.14) | 3.77 (0.16) |  |
| Control |  |  |  |  |
| Male |  | 3.73 (0.18) | 3.91 (0.20) |  |
| Female |  | 3.76 (0.16) | 3.89 (0.17) |  |
| *Depression* |  |  |  |  |
| Intervention | 0.63 |  |  | *F*(1, 458.87) = 0.00*, p* = .923 |
| Male |  | 0.63 (0.04) | 0.66 (0.05) |  |
| Female |  | 0.65 (0.04) | 0.70 (0.04) |  |
| Control |  |  |  |  |
| Male |  | 0.57 (0.04) | 0.66 (0.05) |  |
| Female |  | 0.63 (0.04) | 0.76 (0.05) |  |
| *Anxiety* |  |  |  |  |
| Intervention | 0.63 |  |  | *F*(1, 467.52) = 0.98*, p* = .322 |
| Male |  | 0.61 (0.04) | 0.63 (0.05) |  |
| Female |  | 0.64 (0.03) | 0.60 (0.04) |  |
| Control |  |  |  |  |
| Male |  | 0.61 (0.04) | 0.67 (0.04) |  |
| Female |  | 0.61 (0.04) | 0.70 (0.04) |  |
| *Self-Compassion* |  |  |  |  |
| Intervention | 3.96 |  |  | *F*(1, 469.56) =0.00 *, p* = .95. |
| Male |  | 4.05 (0.04) | 4.05 (0.04) |  |
| Female |  | 3.96 (0.04) | 3.94 (0.04) |  |
| Control |  |  |  |  |
| Male |  | 4.02 (0.04) | 3.97 (0.04) |  |
| Female |  | 3.94 (0.04) | 3.87 (0.04) |  |
| *Well-being* |  |  |  |  |
| Intervention | 3.57 |  |  | ***F*(1,467.18) = 5.74*, p* = .021** |
| Male |  | 3.57 (0.05) | 3.49 (0.06) |  |
| Female |  | 3.54 (0.04) | 3.54 (0.06) |  |
| Control |  |  |  |  |
| Male |  | 3.60 (0.05) | 3.62 (0.06) |  |
| Female |  | 3.60 (0.05) | 3.42 (0.05) |  |
| *Intrinsic Motivation* |  |  |  |  |
| Intervention | 4.81 |  |  | ***F*(1, 462.35) = 4.48, *p* = .041** |
| Male |  | 4.79 (0.09) | 4.58 (0.11) |  |
| Female |  | 4.62 (0.08) | 4.60 (0.09) |  |
| Control |  |  |  |  |
| Male |  | 4.75 (0.09) | 4.75 (0.10) |  |
| Female |  | 4.81 (0.09) | 4.59 (0.10) |  |

*Note.* Higher scores indicate poorer outcomes for depression, anxiety, better outcomes for well-being, self-compassion, intrinsic motivation and greater perfectionism. Significant effects are bolded.

Supplementary Table 2: *Adjusted Means and Standard Errors Across Time and Interactions with Condition, Time and Perfectionistic Concerns Level (High, Low).*

| Outcome measure | Baseline covariate  (*M*) | Post-intervention  *M* (*SE*) | 3-month follow-up  *M* (*SE*) | Condition x time x perfectionism level |
| --- | --- | --- | --- | --- |
| *High Standards* |  |  |  |  |
| Intervention | 5.45 |  |  | *F*(1,477.93) = 0.01*, p* = .932 |
| Low |  | 5.16 (0.09) | 5.06 (0.10) |  |
| High |  | 5.31 (0.07) | 5.17 (0.08) |  |
| Control |  |  |  |  |
| Low |  | 5.35 (0.09) | 5.22 (0.10) |  |
| High |  | 5.34 (0.08) | 5.14 (0.09) |  |
| *Perfectionistic Concerns* |  |  |  |  |
| Intervention | 3.93 |  |  | *F*(1, 453.13) = 1.48*, p* = .233 |
| Low |  | 3.68 (0.10) | 3.92 (0.09) |  |
| High |  | 3.94 (0.10) | 3.76 (0.12) |  |
| Control |  |  |  |  |
| Low |  | 3.88 (0.10) | 3.99 (0.12) |  |
| High |  | 3.98 (0.10) | 3.92 (0.10) |  |
| *Perfectionistic Strivings* |  |  |  |  |
| Intervention | 3.77 |  |  | ***F*(1, 295.33) = 4.24*, p* = .041** |
| Low |  | 4.20 (0.11) | 3.88 (0.11) |  |
| High |  | 3.35 (0.12) | 3.70 (0.12) |  |
| Control |  |  |  |  |
| Low |  | 3.70 (0.12) | 3.73 (0.12) |  |
| High |  | 4.23 (0.12) | 4.44 (0.12) |  |
| *Depression* |  |  |  |  |
| Intervention | 0.63 |  |  | *F*(1, 462.37) = 1.68*, p* = .201 |
| Low |  | 0.59 (0.04) | 0.55 (0.04) |  |
| High |  | 0.67 (0.04) | 0.80 (0.04) |  |
| Control |  |  |  |  |
| Low |  | 0.53 (0.04) | 0.62 (0.05) |  |
| High |  | 0.67 (0.04) | 0.81 (0.05) |  |
| *Anxiety* |  |  |  |  |
| Intervention | 0.63 |  |  | *F*(1, 473.38) = 1.38*, p* = .243 |
| Low |  | 0.56 (0.04) | 0.45 (0.04) |  |
| High |  | 0.71 (0.04) | 0.73 (0.04) |  |
| Control |  |  |  |  |
| Low |  | 0.51 (0.04) | 0.59 (0.04) |  |
| High |  | 0.71 (0.04) | 0.79 (0.04) |  |
| *Self-Compassion* |  |  |  |  |
| Intervention | 3.96 |  |  | *F*(1, 468.61) = 1.11*, p* = .29 |
| Low |  | 4.04 (0.04) | 4.00 (0.05) |  |
| High |  | 3.98 (0.04) | 4.00 (0.04) |  |
| Control |  |  |  |  |
| Low |  | 4.02 (0.05) | 3.97 (0.05) |  |
| High |  | 3.95 (0.04) | 3.88 (0.04) |  |
| *Well-being* |  |  |  |  |
| Intervention | 3.57 |  |  | *F*(1, 469.60) = .69*, p* = .413 |
| Low |  | 3.59 (0.05) | 3.64 (0.05) |  |
| High |  | 5.53 (0.04) | 3.43 (0.05) |  |
| Control |  |  |  |  |
| Low |  | 3.68 (0.05) | 3.63 (0.05) |  |
| High |  | 3.52 (0.05) | 3.41 (0.05) |  |
| *Intrinsic Motivation* |  |  |  |  |
| Intervention | 4.81 |  |  | *F*(1, 465.80) = 2.32*, p* = .135 |
| Low |  | 4.69 (0.08) | 4.73 (0.10) |  |
| High |  | 4.69 (0.08) | 4.50 (0.09) |  |
| Control |  |  |  |  |
| Low |  | 4.89 (0.09) | 4.75 (0.10) |  |
| High |  | 4.67 (0.09) | 4.58 (0.10) |  |

*Note.* Higher scores indicate poorer outcomes for depression, anxiety, better outcomes for well-being, self-compassion, intrinsic motivation and greater perfectionism on all perfectionism measures. Significant effects are bolded.
